# HIV-Testing Behavior and Associated Factors Among MSM in Chongqing, China: Results of 2 Consecutive Cross-Sectional Surveys From 2009 to 2010

**DOI:** 10.1097/MD.0000000000000124

**Published:** 2014-12-12

**Authors:** Xuefeng Li, Guohui Wu, Rongrong Lu, Liangui Feng, Wensheng Fan, Yan Xiao, Zheya Sun, Heng Zhang, Hui Xing, Yiming Shao, Yuhua Ruan

**Affiliations:** From the State Key Laboratory for Infectious Disease Prevention and Control, National Center for AIDS/STD Control and Prevention, Chinese Center for Disease Control and Prevention, Collaborative Innovation Center for Diagnosis and Treatment of Infectious Diseases, Beijing (XL, YX, ZS, HZ, HX, YS, YR); Karamay Center for Disease Control and Prevention, Karamay (XL); Chongqing Center for Disease Control and Prevention, Chongqing, P.R. China (GW, RL, LF); and Department of Public Health, College of Health and Human Services, Western Kentucky University, Bowling Green, Kentucky (WF).; ∗ Drs Xuefeng Li, Guohui Wu, and Rongrong Lu contributed equally to the writing of this article.

## Abstract

The high and climbing human immunodeficiency virus (HIV) rates among Chinese men who have sex with men (MSM) bring huge pressure and challenge to acquired immune deficiency syndrome response work in China. This study examined HIV-testing behavior and describes the characteristics of recently tested MSM in Chongqing to address targeting HIV prevention interventions.

Two consecutive cross-sectional surveys were conducted among Chongqing MSM using respondent-driven sampling in 2009 and 2010. Information was collected regarding details on demographic characteristics, sexual practices with male and female partners, and HIV-testing experiences. Univariate and multivariate logistic regression analyses were performed to identify factors independently associated with recent HIV testing.

The final sample size included in our analyses was 992. The overall HIV prevalence was 13.4%, and HIV prevalence increased significantly from 11.6% in 2009 to 15.4% in 2010 (*P* = 0.08). The overall rate of HIV testing in the past 12 months was 44.6%, and the self-reported rates decreased significantly from 47.8% in 2009 to 41.1% in 2010 (*P* = 0.03). Factors independently associated with recent HIV testing included living in Chongqing >1 year (adjusted odds ratio [AOR] 1.8, 95% confidence interval [CI] 1.1–2.9), the age of most recent male partner ≤25 (AOR 1.5, 95% CI 1.1–2.1), not having unprotected insertive anal sex with most recent male partner in the past 6 months (AOR 1.5, 95% CI 1.1–2.0), disclosing HIV status to most recent male partner (AOR 2.8, 95% CI 2.0–3.8), and holding lower level of HIV-related stigma (AOR 1.1 per scale point, 95% CI 1.0–1.1).

The extremely high HIV prevalence and low annual testing level put MSM at high risk of HIV infection and transmission, and it is a priority to promote regular HIV testing among this group in order to control the spread of HIV in Chongqing, China.

## INTRODUCTION

The high and climbing human immunodeficiency virus (HIV) rates among Chinese men who have sex with men (MSM) bring huge pressure and challenge to acquired immune deficiency syndrome (AIDS) response work in China. The recent national HIV sentinel surveillance data reported that HIV prevalence among Chinese MSM increased significantly from 0.9% in 2003 to 6.3% in 2011, which is far above the general population level (0.058% in 2011).^1,2^ In addition to these figures, transmission modes estimated that 32.5% of all new infections in 2009 and 29.4% in 2011 in China were attributed to homosexual transmission.^3^ Thus, MSM remain an important driver of Chinese HIV/AIDS epidemic.

The 2012 China AIDS response progress report indicated that large number of HIV-infected persons in China remained unaware of their infection, constituting a risk for further transmission. A mathematical model estimated that 87% of all HIV cases among Chinese MSM remained undiagnosed.^2,4^ Previous research estimated that people who were HIV positive but unaware of the condition are 3.5 times more likely to transmit HIV compared with diagnosed people.^5^ Regular HIV testing has been found to be a highly effective strategy for controlling the HIV/AIDS epidemic. Frequent testing allows early diagnosis and creates opportunities for early linkage to medical care and treatment, knowledge of HIV status can help motivated people change risky sexual behaviors to reduce further transmission to others.^6–9^

United States and Australia guidelines both recommend annual testing for sexually active MSM and every 3 to 6 months for those with particularly high-risk behaviors.^10,11^ The Chinese plan for HIV/AIDS prevention and control among MSM (2007–2010) called for the whole society work together to combat the HIV epidemic among MSM; 85% of local MSM were provided with HIV counseling and 60% of those counseled were tested for HIV by the end of 2010. However, the plan just called for increasing the coverage without setting the frequency of testing.^12^

We conducted 2 cross-sectional surveys to examine the testing behavior and the correlation of recent HIV testing among Chongqing MSM during 2009 to 2010. Recent HIV testing was defined as last testing within the previous year before the interview. The objective of this study is to identify the characteristics of men who lack regular testing and address targeting HIV prevention programs to enhance HIV testing among this high-risk group.

## METHODS

### Participants and Procedures

Two consecutive cross-sectional surveys were conducted among Chongqing MSM using respondent-driven sampling (RDS). The participants were recruited from October to December in 2009, and from September to December in 2010. The recruitment and interview were conducted at the sexually transmitted disease (STD) clinic of Chongqing Centers for Disease Control and Prevention, and the main responsibility of the clinic is to provide voluntary counseling and testing. The target population for this survey was MSM who age 18 years or older, had sex with another man in the past 12 months (oral, anal, or mutual masturbation), currently living in Chongqing, and had a valid recruitment coupon (to have a thoughtful details of our survey a coupon was given to recruit the new participants into the survey).

We employed RDS to recruit participants. This method has been described extensively.^13,14^ In summary, “seeds” were selected to initiate the recruitment, they were given 3 recruitment coupons to refer their network peers to the study, and these people, in turn, refer up to 3 other peers, and so on until a desired sample size was achieved. The information printed on the coupons mainly included a unique study identification code which can link each participant to his recruiter, the phone number of the interview site, the address, and a map to the project site. Selected seeds were different in terms of geographic, demographic, and other key variables. In our study, 7 and 6 seeds were selected in 2009 and 2010, respectively. Eligible seeds and recruits were consented, enrolled, interviewed, and blood was collected for serological testing. Both a primary incentive (30 RenMinBi for completing the survey) and a secondary incentive (20 RMB for recruiting each eligible participant) were given to participants. Pre- and posttest counseling was provided to all the participants, and healthcare and social services were offered to people as needed. Participants could obtain the test result by face-to-face consultation or by telephone within 1 to 3 weeks.

Having MSM participate only once in the survey is crucial to ensuring unbiased estimates and the highest quality of data. We took up the following to assist in detecting potential duplicate enrollments: participant-recruiters were instructed that they should only distribute coupons to persons who have not yet participated in the current study; the same staff worked during screening and interview hours to increase the chance of recognizing men who have already participated; and study site maintained a coupon log book for daily operations (eg., date, coupon numbers of persons enrolling, coupon numbers of persons who agree to recruit, etc) and to record distinguishing features of participants (eg, glasses, earrings, tattoos, scars, etc).

The estimated proportion of recent testing rate at the time of survey determined the sample size (for example, we used recent testing rate at an estimated 30% according to one recent survey of MSM in China).^15^ Based on the formula of sample size, we needed 400 MSM participants per survey year; therefore, we increased up to a targeted 500 to account for potential missing data. In addition to meeting the sample size, RDS requires achieving equilibrium (ie, stabilization of the variables in the cumulative sample); we therefore tracked equilibrium for several key demographic and risk variables during recruitment to ensure that 500 MSM and equilibrium is achieved.

### Measures

Each participant completed a self-administered computer-assisted interview. Information was collected regarding details on demographic characteristics, sexual practices with male and female partners, and HIV-testing experiences.

In terms of HIV-testing history, all participants were asked to recall the date and result of their most recent HIV test. Recent testing was defined as last testing within the previous year before the interview.

Two psychosocial scales which have been proved to be appropriate for use among Chinese MSM were adapted in our study.^16^ We measured stigma and discrimination attitudes toward HIV/AIDS by 22 agree/disagree (1 = “yes”, 2 = “no”) statements. This scale included shame/blame/isolation subscale, perceived discrimination subscale and equity subscale, and included questions such as “People with AIDS should be isolated from other people.” Items were summed to create total scale scores (range = 22–44, Cronbach alpha 0.75) with a higher score indicating a lower level of HIV-related stigma. Attitude toward safer sex was a scale (Cronbach α 0.80) constructed from 15 items (each scored from 0 = strongly disagree to 4 = strongly agree) such as “I am able to avoid behavior that may put me at risk of HIV infection.” In our analysis, a higher total score indicated a higher level of positive attitudes and perceptions of abilities for safe sex.

### Data Analysis

During the analysis, we pooled the data from 2 waves, and excluded MSM who participated in the survey repeatedly (repeated participants were judged by self-reported data and by comparing their features, such as telephone number, QQ number, birthdate, etc). We employed chi-square test to compare sample demographic and behavior characteristics across survey years. Univariate and stepwise multivariate logistic regression analyses were performed to identify factors independently associated with recent HIV testing. Factors that were statistically significant (*P* < .05) in univariate analyses were considered candidates for inclusion in the multivariate model. Considering that respondent-driven sampling analysis tool (RDSAT) could not perform multivariate analysis,^17^ and the data from the 2 waves were combined (the 2 networks were distinct and not linked, RDSAT weights were not applied to the pooled data),^18^ we did not use weighted RDS analysis. All analyses were conducted using SAS version 9.1 (SAS Institute; Cary, NC, USA), and the significance level was set at *P* < 0.05.

### Laboratory Methods

Specimens were tested for HIV and syphilis antibody. HIV antibodies were screened using enzyme-linked immunoassay (Vironostika HIV Uni-Form plus O, bioMerieux, Holland). If the result was positive, a western blot test was conducted for confirmation (HIV Blot 2.2 WB, MP Biomedicals Co, Ltd., Singapore). Syphilis screening was performed by rapid plasma reagin (Shanghai Rongsheng, China) and confirmed by the treponema pallidum particle assay ( Fujirebioinc, Japan). MSM were considered current syphilis infected if both tests were positive.

### Ethics Statement

The study protocol was developed and approved by the institutional review boards of the National Center for AIDS/STD Control and Prevention in China, Vanderbilt University, and the University of California, San Francisco. All participants gave informed consent before the interview.

## RESULTS

### Sample Characteristics

A total of 510 participants in 2009 and 498 participants in 2010 were recruited and completed the survey. In the second wave, 16 MSM participated again from the first wave. As noted previously, 16 repeated participants were removed from the second sample, and data from the 2 waves were pooled together for the analyses of associated factors of recent HIV testing.

Table 1  presents the characteristics of the sample by survey year. Comparison of sample characteristics for the 2 waves revealed that major variables were similar. Across the survey years, more MSM who were >25 years old or had health insurance were recruited. The number of MSM who reported drinking alcohol before/during sex with last male partner decreased from 50.2% in 2009 to 39.2% in 2010 (*P* = 0.001), whereas the number of seeking last male partner through the internet increased from 70.9% in 2009 to 79.0% in 2010 (*P* = 0.004). A greater proportion reported HIV-status disclosure to or from most recent male partner in 2010 than 2009, which increased from 33.3% in 2009 to 41.1% in 2010 (*P* = 0.01), and from 24.6% to 31.1% (*P* = 0.03), respectively. In terms of female partner, a slightly higher proportion of MSM married (8.1% vs 5.7%) ever had sex with a women (39.7% vs 39.4%), and had female sexual partner in the past 6 months (11.0% vs 7.8%) in 2010 compared with 2009, but there were no significantly differences between the 2 waves. There was no significant increase in HIV prevalence, that is 11.6% in 2009 to 15.4% in 2010 (*P* = 0.08) and the overall HIV prevalence was 13.4%.

**TABLE 1:**
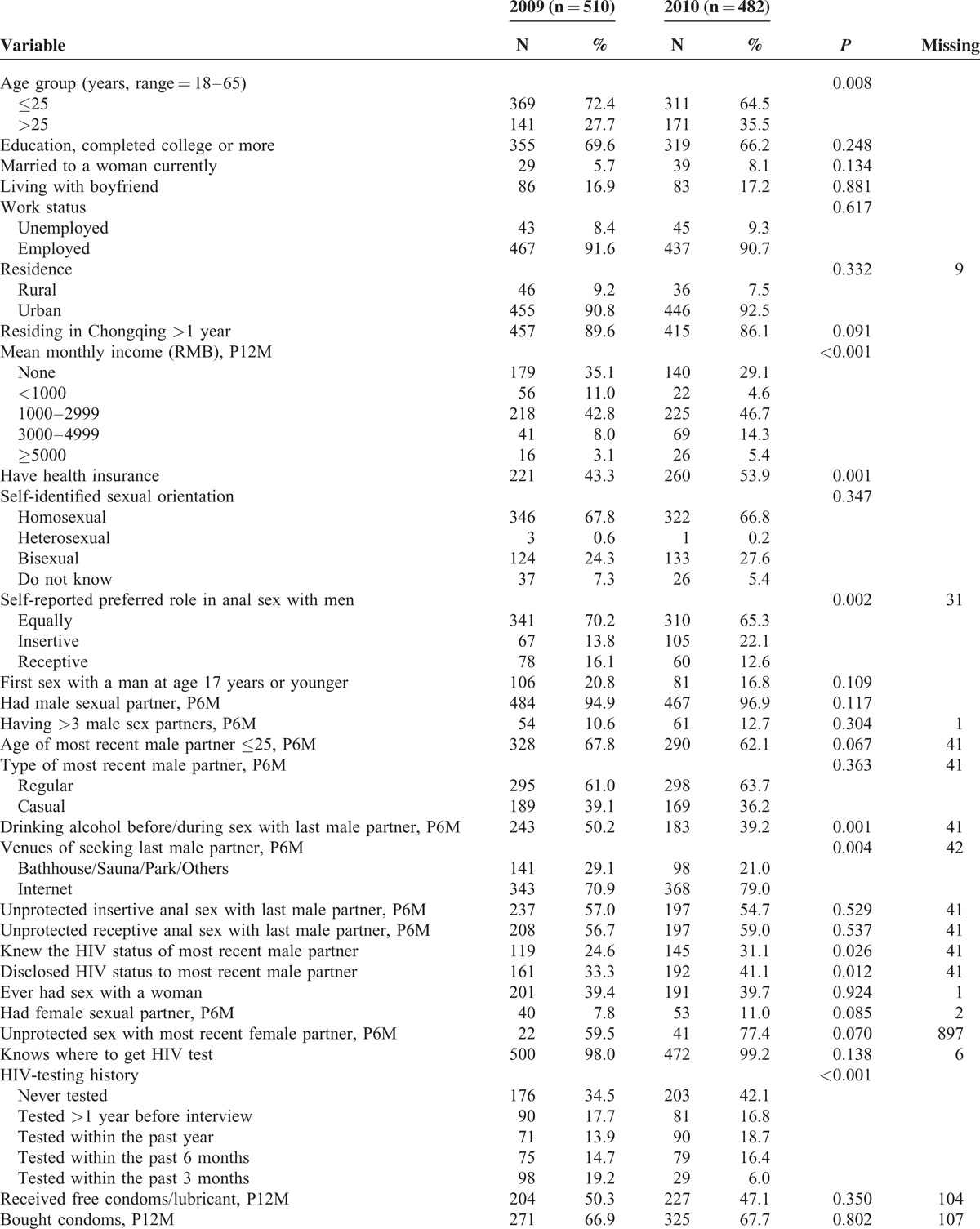
Comparisons of Demographic and Behavior Characteristics by Survey Year Among MSM in Chongqing, China, 2009–2010 (N = 992)

**TABLE 1 (Continued):**
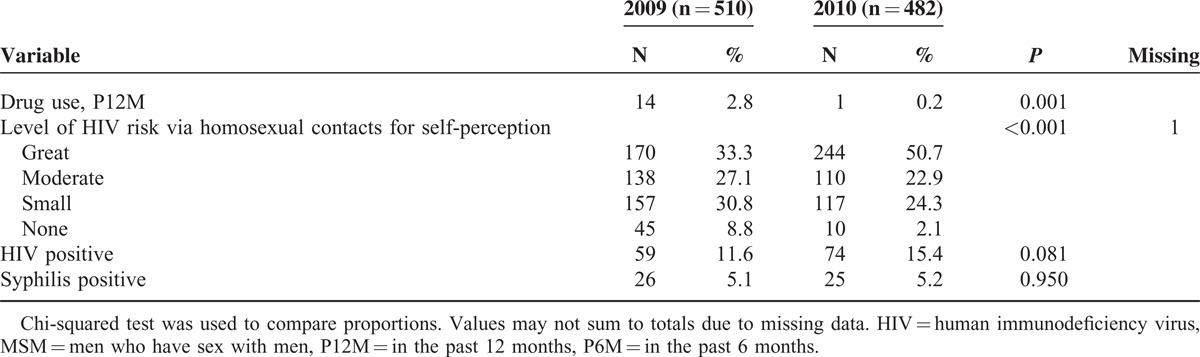
Comparisons of Demographic and Behavior Characteristics by Survey Year Among MSM in Chongqing, China, 2009–2010 (N = 992)

### HIV Test and HIV Status

With respect to the HIV-testing history of the 992 participants, 127 (12.8%) had last tested ≤3 months before the interview, 154 (15.5%) ≤6 months, 161 (16.2%) ≤12 months, 171 (17.2%) >12 months, and 379 (38.2%) had never been previously tested. The overall lifetime testing rate was 61.8% with 65.5% in 2009, 57.9% in 2010 (*P* = 0.01). The overall rate of HIV testing in the past 12 months was 44.6% with 47.8% in 2009, 41.1% in 2010 (*P* = 0.03).

Among 442 MSM who self-reported HIV testing in the past 12 months, 40 (9.1%) were tested HIV positive in this study, whereas from 550 who reported not testing in the past 12 months, 93 (16.9%) were tested positive (*P* < 0.001).

Of note, among 133 MSM who tested positive through our study, only 2 (1.5%) people were previously diagnosed, and 131 (98.5%) were unaware of their HIV infection at the time of interview (38 MSM perceived themselves to be HIV negative and 93 self-reported of not knowing their serostatus).

### Correlates of Recent HIV Testing

Table 2  describes the univariate and multivariate logistic analyses of variables in relation to recent HIV testing among Chongqing MSM. Factors that were statistically significant (*P* < 0.05) in univariate analyses were included in the multivariate model and selected by stepwise method, the variables included age, education level, residence, years residing in Chongqing, self-identified sexual orientation, self-reported preferred role in anal sex with men, age of most recent male partner in the past 6 months, drinking habit, unprotected insertive anal sex with last male partner in the past 6 months, HIV-status disclosure to or from most recent male partner, having female sexual partner in the past 6 months, self-perceived level of HIV risk via homosexual contacts, and holding lower level of HIV-related stigma. In the final multivariable model, factors independently associated with recent HIV testing included living in Chongqing >1 year (AOR 1.8, 95% CI 1.1–2.9), the age of most recent male partner ≤25 (AOR 1.5, 95% CI 1.1–2.1), not having unprotected insertive anal sex with most recent male partner in the past 6 months (AOR 1.5, 95% CI 1.1–2.0), disclosing HIV status to most recent male partner (AOR 2.8, 95% CI 2.0–3.8), and holding lower level of HIV-related stigma (AOR 1.1 per scale point, 95% CI 1.0–1.1).

**TABLE 2:**
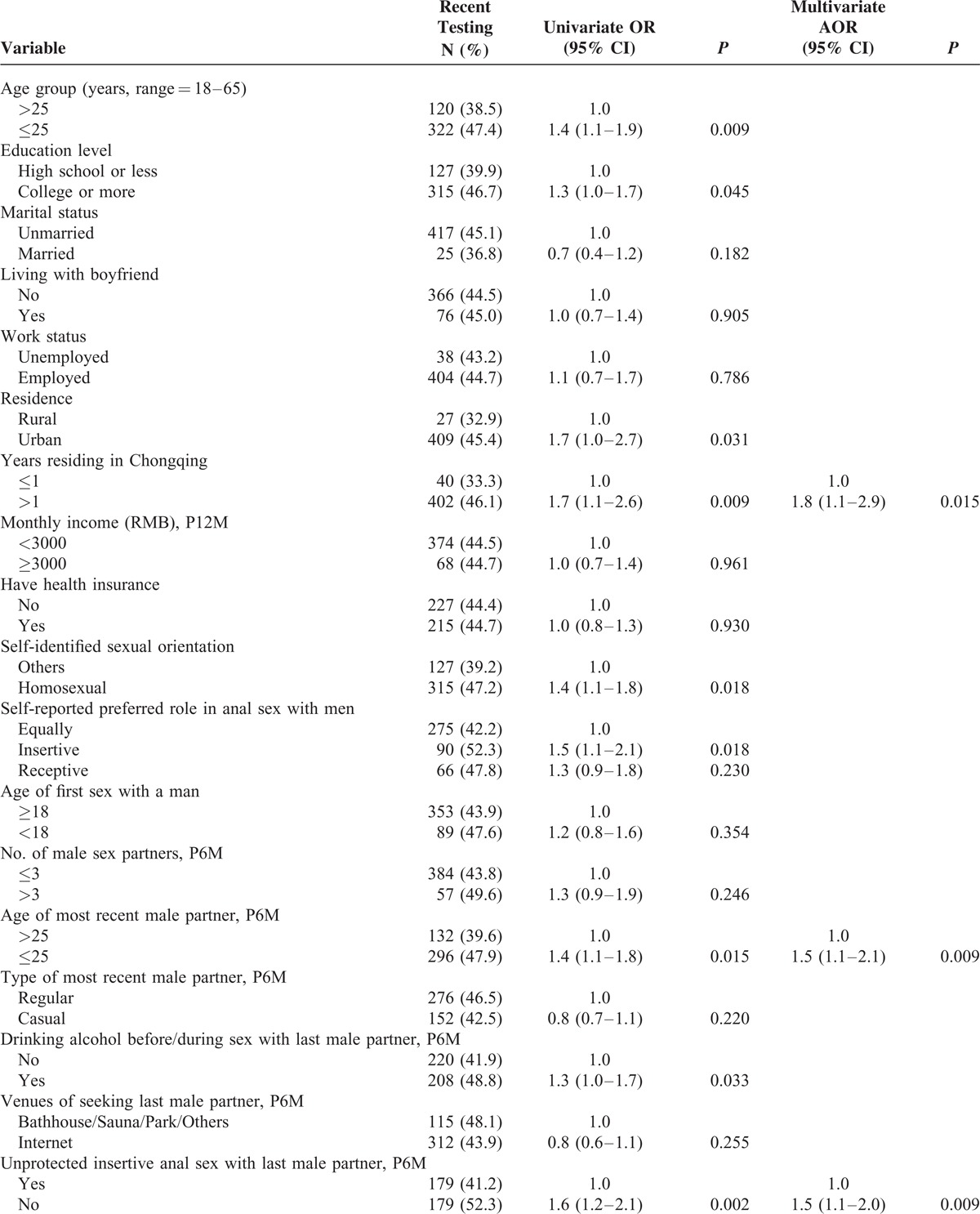
Factors Associated With Recent HIV Testing Among MSM in Chongqing, China, 2009–2010 (N = 992)

**TABLE 2 (Continued):**
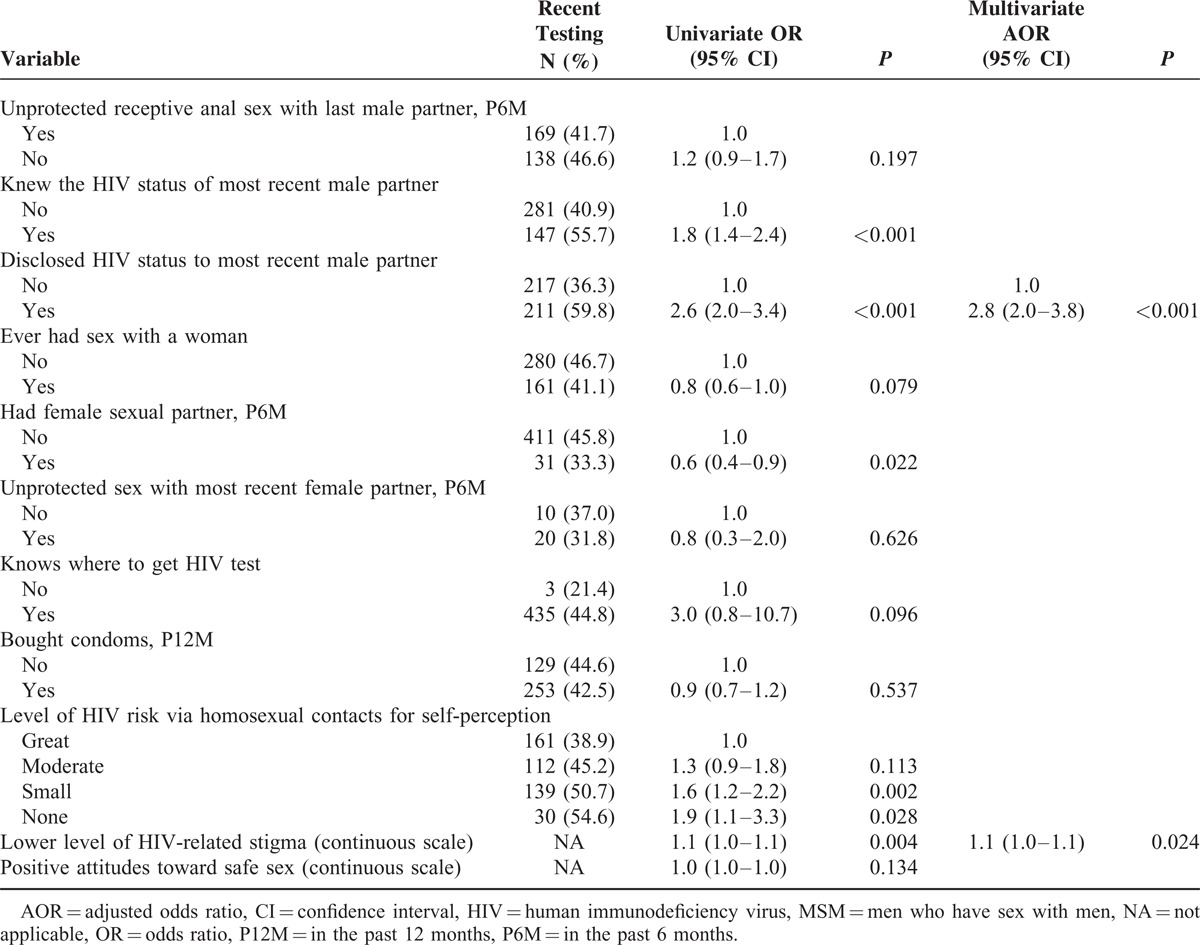
Factors Associated With Recent HIV Testing Among MSM in Chongqing, China, 2009–2010 (N = 992)

## DISCUSSION

According to our data, HIV prevalence remained high among Chongqing MSM and these MSM lack frequent HIV testing. The study also found MSM living in Chongqing >1 year, the age of most recent male partner ≤25, not having unprotected insertive anal sex with most recent male partner in the past 6 months, disclosing HIV status to most recent male partner, and holding lower level of HIV-related stigma were independently associated with recent HIV testing.

The severe HIV infection status among MSM causes a great public health concern in China. A recent meta-analysis indicated that the rates of HIV infection of Chinese MSM varied significantly across geographic locations and Chongqing reported the highest HIV infection rate with 12.5% in 2007.^19^ Also, an RDS sample of 617 MSM in Chongqing in 2008 reported 16.8% of the participants were HIV positive.^20^ Our study found similar HIV prevalence among Chongqing MSM, increased from 11.6% in 2009 to 15.4% in 2010. These figures far surpassed the national HIV sentinel surveillance data, which is 6.3% in 2011 among Chinese MSM.^1^

Our study demonstrates both lifetime testing and recent testing rates are unacceptably low. The overall rate of HIV testing in the past 12 months was 44.6% which decreased from 47.8% in 2009 to 41.1% in 2010. The recent testing rate was found to be somewhat lower than the national annual HIV-testing rate, which was 50.4% among Chinese MSM in 2011.^2^ The annual testing level of Chongqing MSM is also far below the rate that is reported in many developed countries (60%–70% in Australia^21,22^ and 67% in the USA in 2011^23^). Of note, MSM who did not test in the past 12 months were almost twice as likely to be tested HIV positive in our study compared with men who recently tested (16.9% vs 9.1%), and almost all HIV positive men (98.5%) were unaware of their HIV infection at the time of interview. With respect to low testing rate and increased number of HIV-infected MSM, unaware of their infection, we examined the associations of recent HIV testing among Chongqing MSM using the pooled data during 2009 to 2010 in order to design targeting interventions.

We found that MSM who lived for ≤1 year in Chongqing were less likely to be tested in the past 12 months. A study conducted among Beijing migrant MSM in 2009 noted that this group might have been more vulnerable to the risk of HIV due to low socioeconomic status, low consistent condom use, and limited knowledge of the benefits of early HIV diagnosis and treatment.^24,25^ This strongly suggests that more attention should be paid to recent migrants to the city. It is worth noting that most of our participants lived in Chongqing >1 year with low testing, highly unknown HIV status levels, it is advantageous for all MSM to receive tailored prevention programs. Preventions should focus on increasing their knowledge about HIV/AIDS and early testing benefits and increasing their awareness of HIV risk, in order to increase the frequency of HIV testing.

In our study, MSM with a most recent male partner ≤25 years of age were more likely to be recently tested. Previous studies have also reported that younger age was associated with more frequent HIV testing^21,26–28^; younger MSM may be more sexually active and have more extensive social networks, thus may be more acceptable with new things and more likely to receive regular HIV testing.^29^ Future health interventions should target older men and promote peer education to increase their testing behaviors.

Previous researches have shown different findings regarding the relationship between HIV testing and high-risk sexual behaviors, some reported that MSM who had unprotected anal intercourse were more likely to undergo testing because they might be aware of their increased risk for HIV infection and HIV testing may be seen as a tool to assess the possibility of transmission,^21,24,30^ whereas others reported the opposite result.^18,31–33^ Consistent with later result, we also found that MSM who engaged in unprotected insertive anal sex with most recent male partner were less likely to get tested in the previous 12 months. The proportions of men engaged in unprotected insertive or receptive anal sex with last male partner in our study were between 55% and 60% in both years. The high rates of unprotected anal sex was similar to those reported by other places in China, such as 62.3% in Nanjing in 2008, 60.3% in Guangzhou in 2008, and 68.7% in Shandong in 2011.^34–36^ In our study, men who are less likely to have sex without condom may be more likely to test for HIV, which might be because they are the “worried well” – those who are exceptionally conscientious of their health status. On the contrary, men who engage in high-risk sexual practices may lack the awareness of HIV infection risk and lack effective HIV-testing interventions. Hence, it is essential to promote safer sex and increase testing coverage among this subgroup.

Our result of the association between disclosing HIV status to most recent male partner and recent HIV testing is consistent with previous findings, which indicated that HIV disclosure to or from male partners was a significant predictor of recent HIV testing.^21,30,37^ Several studies have found that HIV status disclosure may reduce the risk of HIV transmission by reducing risky sexual behaviors.^38,39^ Thus, disclosure is a key to prevention strategy. HIV-infected but unaware MSM might send the wrong message; hence, it is urgently needed to promote not only regular HIV testing but also accurate HIV disclosure.

Studies have consistently indicated that HIV-related stigma could influence the uptake of HIV-testing services, HIV disclosure, and the effectiveness of care and treatment.^16,40–42^ Like previous research, we observed that MSM who reported lower levels of stigma and discrimination attitudes toward HIV/AIDS were more likely to be recently tested. HIV-related stigma is common in China. The general population may believe that punishment was an appropriate response toward those living with HIV,^43^ a majority of service providers may have negative attitudes toward people with HIV and have the perception of being stigmatized due to working with HIV-positive people.^44–46^ In our study, MSM reported internalized HIV-related stigma, which poses a great challenge for this marginalized population, highlights the need to strengthen the education of HIV/AIDS, reduce internalized and external HIV stigma, and enhance HIV testing among this group by accelerating each other. Decreased stigma may increase testing, whereas increased testing on the contrary could result in greater acceptance and reduce stigma.^47^

There are several limitations to this study. First, the questionnaire gathered sensitive information regarding sexual behavior and the history of alcohol and drug use, participants may tend to give social desirable responses, such as underreporting risky sexual behavior or substance use, which might affect our estimates. Second, considering our survey was cross-sectional, we cannot determine the causality of the statistically significant associations with recent HIV testing. Third, we did not use RDSAT weights, and hence the estimates may be biased by over- or underrepresented subgroups of the population (such as lower socioeconomic persons might be more likely to participate in the survey due to monetary incentive), and limit the external validity of our conclusions.^48^ Fourth, HIV testing was originally part of the study design, individuals who were less likely to get tested might also be less likely to participate in our survey, which may result in overestimation of the actual HIV-testing levels. Finally, there are only 2 waves of data; thus, it is important to note that changes in the frequencies of variables over time are not necessarily reflective of actual trends. Three time points are needed for a trend analysis, so further research is needed to explore the actual changing trends of risk behaviors among MSM.

Despite the above limitations, this study indicates that extremely high HIV prevalence and low annual testing level put Chongqing MSM at high risk of HIV infection and transmission. According to the characteristics and needs of the target population, future HIV prevention and intervention programs should continue to educate MSM about HIV/AIDS knowledge, behavioral risks, benefits of early testing, encourage disclosure, and reduce HIV-related stigma. This scaling-up HIV-testing efforts among Chongqing MSM is vital with respect to the efficacy of HIV-status-dependent HIV interventions. When promoting regular testing, men who are older, recent migrants to the city, nondiscloser, reporting unprotected anal sex, and men with higher level of HIV-related stigma are worthy of our attention. HIV and AIDS can pose a serious threat to human health, social stability, and economic development of the society. Therefore, the government plays an important role in calling for the whole society to work together to prevent and control HIV/AIDS, and the government's policy is of great significance. It is increasingly a priority for Chinese government to make specific guidelines regarding testing frequency for high-risk populations like developed countries in order to early identify and treat patients, and ultimately achieve the goal of strongly curbing and minimizing the spread of AIDS.
